# Effect of sodium bicarbonate on performance and markers of kidney and gastrointestinal injury during repeated sprints in the heat

**DOI:** 10.1007/s00421-026-06266-x

**Published:** 2026-05-14

**Authors:** Jonathan W. Specht, Marissa Ramirez, Jennifer Raef, Mackenzie Schwartz, Alyssa R. Bailly, Oscar Chavez, Ozan Kumbasar, Jade X. Dawson, Jessica Apodaca, Raul Freire, Whitley C. Atkins, Flávio de Castro Magalhães, Jason Siegler, Fabiano T. Amorim

**Affiliations:** 1 Department of Kinesiology and Athletic Training, University of Northern Iowa, WRC 139, Cedar Falls, IA 50613 USA; 2 Department of Health, Exercise, and Sports Sciences, University of New Mexico, Albuquerque, NM USA; 3 Department of Exercise and Nutrition Sciences, Center for Research and Education in Special Environments, University at Buffalo, Buffalo, NY USA; 4 Marmara University, Istanbul, Turkey; 5 College of Health Solutions, Arizona State University, Phoenix, AZ USA

**Keywords:** Endotoxemia, Ergogenic aids, Intervals, Repeat sprint ability

## Abstract

**Purpose:**

We tested the hypotheses that sodium bicarbonate (SB) supplementation improves performance, reduces markers of acute kidney injury (AKI) risk, and increases markers of acute gastrointestinal injury (AGI) during repeated sprints in the heat.

**Methods:**

Using a double-blind design, 10 recreationally endurance-trained participants ingested 0.2 g/kg of body weight of SB or placebo before cycling in the heat (dry-bulb: 40 °C, relative humidity: 20%). Exercise bouts included a 22-min warm-up, then 4-sets of 5 × 6-sec maximal-effort standing cycling sprints. Core temperature (Tc) was measured at baseline, end of warm-up, after set two of sprints, and post-exercise bouts. Urine and plasma collected pre-ingestion and pre-, post-, and 1-h post-exercise were analyzed for markers of AKI risk, AGI, and endotoxemia.

**Results:**

SB increased peak (average difference between conditions: 21 ± 11 W, condition effect: *p* = 0.02) and average (*p* = 0.03) power across the sprints. Tc increased from pre- to post-exercise (placebo: Δ1.3 ± 0.3 °C, SB: Δ1.4 ± 0.4 °C, time effect: *p* < 0.001), but there were no effects of condition or interaction. Exercise increased the primary marker of AKI (urinary IGFBP7×TIMP-2, time effect: *p* = 0.005) but not urinary NGAL. There were no condition or interaction effects for either AKI marker. Plasma I-FABP, a marker of AGI, increased post-exercise bouts (placebo: Δ291 ± 550 pg/mL, SB: Δ397 ± 482 pg/mL, *p* = 0.03), with no condition or interaction effects. There were no significant effects for markers of endotoxemia (LBP, sCD14).

**Conclusions:**

SB supplementation improves repeat sprint performance in the heat without altering Tc, markers of AKI risk, or markers of AGI.

## Introduction

Sodium bicarbonate (SB), commonly known as baking soda, is an accessible supplement frequently used by athletes to improve repeated sprint ability (RSA) (Siegler et al. 2016). The ergogenic effect is largely due to buffering extracellular hydrogen ions and reducing metabolic acidosis, both of which contribute to fatigue during repeated bouts of high-intensity exercise (Bishop et al. 2004; Girard et al. 2011; Grgic et al. 2021). However, less is known about the impact of SB supplementation on RSA in hot environmental conditions, as these conditions impair performance across almost all exercise modalities, including intermittent sprints, endurance events, and skill-based activities (Gibson et al. 2020).

Exercise performed under hot conditions increases physiological strain (i.e., heart rate, core temperature) due to competing demands of blood flow to the skin and working muscles (Périard et al. 2021). This strain may shift the limiting factors of RSA from metabolic acidosis to hyperthermia, as elevated muscle and core temperatures reduce RSA (Drust et al. 2005). To our knowledge, only one previous study has investigated the impact of SB on sprint performance in the heat. Mündel (2018) (Mündel 2018) found that SB ingestion prior to two 30-second Wingate tests in the heat (dry bulb: 30 °C, relative humidity: 50%) improved peak power and anaerobic capacity (total work completed) during the second Wingate. However, the protocol involved only two sprints, which may not apply to the majority of sports that require several repeated sprints in an event such as soccer, football, and rugby. Additionally, the short heat exposure probably minimized the increase in core temperature (which was not measured in the study) and heat strain.

Beyond performance decrements, athletes performing repeated sprints in the heat may also carry potential detrimental health risks. For example, hyperthermia and dehydration are associated with increased markers of acute kidney injury (AKI) risk (Chapman et al. 2020). In this context, Houck et al. (2022) found that high-intensity exercise intervals in the heat elicit greater increases in markers of AKI compared to moderate-intensity continuous exercise. In animal models, SB has been shown to be a potential aid in reducing chronic heat exposure-induced AKI. Sánchez-Lozada et al. (2018) found that rats treated with SB in drinking water had less evidence of AKI following 35 days of 1-hour episodes of heat stress and dehydration. However, the therapeutic effect of SB supplementation has not been shown in human models and necessitates further research.

A common side effect of SB supplementation is gastrointestinal (GI) distress, including stomach cramps, bloating, and gas (Kerksick et al. 2018). These symptoms are largely caused by the increased production of carbon dioxide in the stomach, which is produced via rapid neutralization of hydrogen ions by bicarbonate (HCO_3_^−^) (Turnberg et al. 1970). Additionally, intestinal mucosa may be irritated from excessive sodium ingestion (Kahle et al. 2013), and high sodium consumption has been shown to damage the intestinal barrier (Chen et al. 2025; Si et al. 2025). Symptoms of GI distress are reduced when SB is taken in capsules and with a high-carbohydrate meal (Carr et al. 2011); however, less is known about whether SB-related GI distress is associated with acute gastrointestinal injury (AGI). Both exercise and heat stress are known to independently and cumulatively increase symptoms of GI distress, AGI, and translocation of pathogenic bacteria into circulation (i.e., endotoxemia) (van Wijck et al. 2012; Dokladny et al. 2016; Snipe et al. 2018). These responses are dependent on exercise intensity, as greater splanchnic hypoperfusion during high-intensity exercise exacerbates GI ischemia and AGI (Pals et al. 1997; Costa et al. 2020). Indeed, McKenna et al. (2022) demonstrated that high-intensity interval exercise in the heat exacerbated bacterial endotoxin translocation compared to moderate-intensity continuous exercise. Although SB is known to increase GI distress, further research is needed to determine if it exacerbates exercise and heat-induced AGI and endotoxemia.

Based on the preceding evidence, this study aimed to test the hypotheses that SB supplementation (1) improves RSA, (2) attenuates markers of AKI, and (3) exacerbates AGI and endotoxemia during repeated sprints in a hot environment. Findings from this study will help to understand both the ergogenic potential and potential health implications of SB supplementation prior to repeated high-intensity exercise in the heat.

## Methods

### Experimental design

**Fig. 1:**
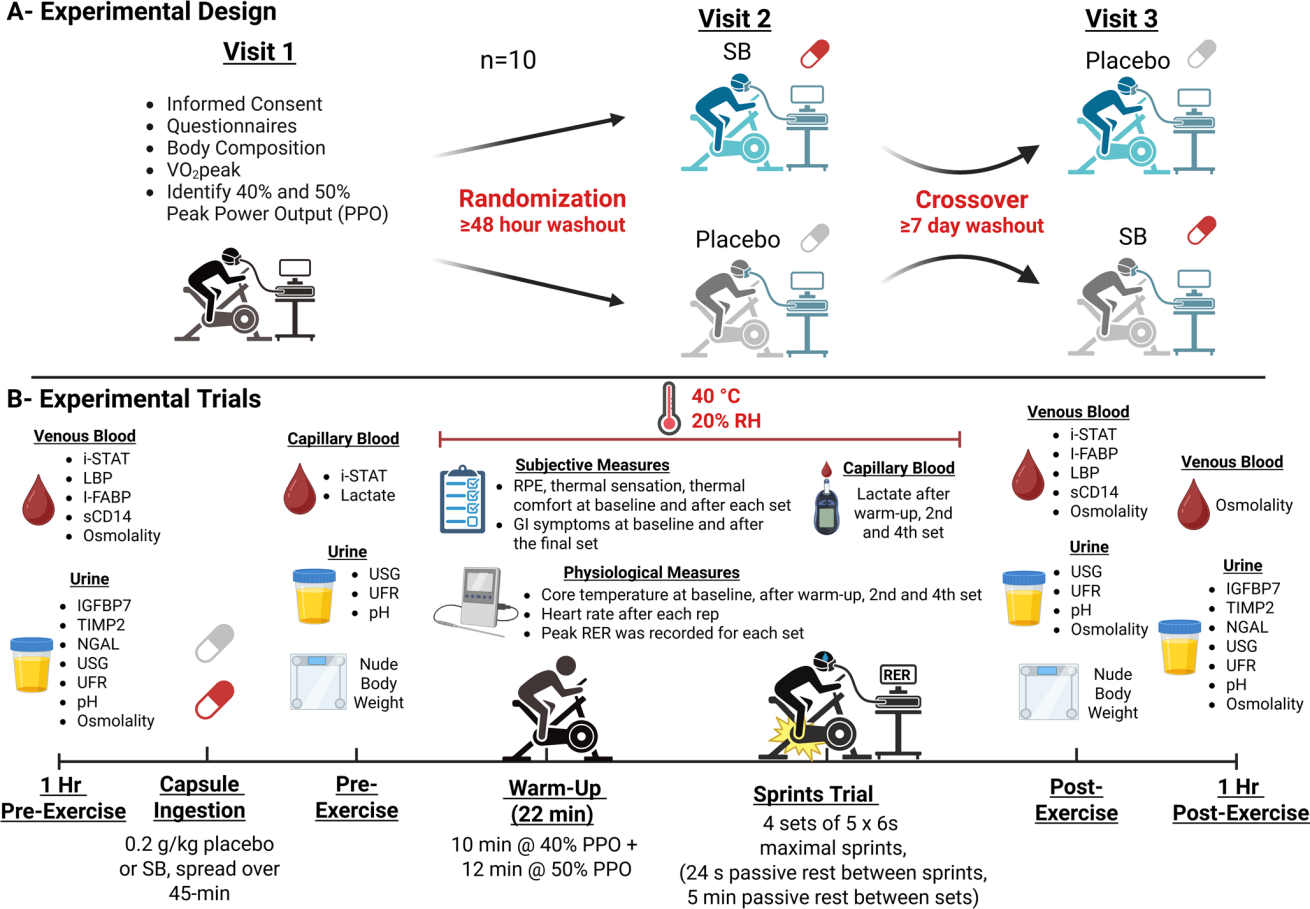
**A** Graphical abstract of experimental design and **B** experimental exercise bout protocol examining the effects of sodium bicarbonate (SB) versus placebo on repeated sprint performance in the heat and markers of acute kidney injury and acute gastrointestinal injury. All measures using the i-STAT device were done using CG8 + cartridges and measured blood bicarbonate concentration, hematocrit, hemoglobin, and pH. I-FABP, intestinal fatty acid binding protein; IGFBP7, insulin-like growth factor-binding protein 7; LBP, lipopolysaccharide binding protein; NGAL, neutrophil gelatinase-associated lipocalin, RER, respiratory exchange ratio; RH, relative humidity; RPE, rating of perceived exertion, sCD14, soluble cluster of differentiation 14; TIMP-2, tissue inhibitor of metalloproteinase 2; UFR, urine flow rate; USG, urine specific gravity; VO_2_peak, peak oxygen consumption. Created with BioRender.com

Participants completed three visits to the Exercise Physiology Laboratory at the University of New Mexico. A graphical experimental design and flowchart for experimental exercise bouts can be found in Fig. 1. During the first visit, participants completed baseline testing, which included paperwork, anthropometric measurements, and a maximal ramped exercise test on a cycle ergometer to determine peak oxygen consumption (V̇O_2_peak). At least 48 h later, visit 2 consisted of the first of two experimental exercise bouts, during which participants completed one hour of exercise in a heat chamber (dry bulb: 40 °C, relative humidity: 20%) after ingesting 0.2 g of SB per kilogram of bodyweight or placebo (corn starch). Participants completed an exercise protocol adapted from Périard et al. (2020), consisting of a 22-minute submaximal warm-up followed by four sets of five, 6-second maximal effort standing sprints on a cycle ergometer. Exercise bouts were conducted using a randomized, counterbalanced, double-blind, crossover design. Participants completed the alternative experimental exercise bouts at least seven days after the initial exercise bout.

### Participant recruitment

Prior to data collection, all procedures were approved by the University of New Mexico institutional review board (Protocol #2402110014). This study was conducted in accordance with the Declaration of Helsinki, with exception of registration. All participants completed an informed consent, health history form, and a physical activity readiness questionnaire (PAR-Q). Inclusion criteria were: ages of 18 and 45 years, self-reported at least 300 weekly minutes of endurance exercise in the last 6 months, and a V̇O_2_max above the 80th percentile for age and sex (American College of Sports Medicine et al. 2021). Exclusion criteria were: history of heat illness, recent repeated heat exposure, signs, symptoms, or known cardiovascular, renal, or metabolic disease, resting blood pressure > 140/90 mmHg, history of regular GI distress, or use of non-steroidal anti-inflammatory drugs one week prior to enrollment.

A required sample size of eight participants was calculated a priori using an alpha level of 0.05, a power of 0.80 (1 − β), assuming a correlation among repeated measures of 0.70, and a partial eta-squared effect size of ηp^2^ = 0.274 (used to calculate an effect size of Cohen’s f = 0.614) (G-Power version 3.1.9.6 software). This effect size was calculated from our previous research, which showed that SB ingestion prior to two hours of walking in the heat reduced markers of AKI (Masoud et al. 2025). The variable used was the product of tissue inhibitor of metalloproteinase 2 (TIMP-2) multiplied by insulin-like growth factor-binding protein 7 (IGFBP7), the only US Food and Drug Administration (FDA) approved marker to predict AKI (Wołyniec et al. 2020).

### Baseline testing

During the initial visit, participants provided informed consent and were screened for eligibility via a health history questionnaire and the PAR-Q. Resting blood pressure was manually measured twice by the same researcher to screen for hypertension. Female participants completed a urine-based pregnancy test and were excluded if the result was positive. Baseline anthropometric assessments (height, weight, body composition) were completed. Height was measured barefoot using a stadiometer (SECA, SECA 216, Chino, Calif., USA), while body weight and percent body fat were measured using a bioelectrical impedance scale (InBody770, InBody USA, Cerritos, CA) with participants wearing shorts and a t-shirt.

After a five-minute warm-up at a self-selected intensity, participants completed a maximal exercise test on an electronically braked cycle ergometer (Lode Excalibur, Groningen, The Netherlands). Testing was done using an individualized ramp protocol (20–30 Watts/min) based on body mass and activity level, designed to elicit volitional exhaustion between 8 and 12 min (Yoon et al. 2007). Expired gases were collected throughout using a metabolic cart (TrueOne 2400, ParvoMedics, Sandy, UT), and heart rate was measured using a chest strap (Polar H10, Kempele, Finland). Peak oxygen consumption (V̇O_2_peak) was determined as the highest V̇O_2_ value averaged over 8-breaths, maximal heart rate as the highest recorded value, and maximal workload as the highest intensity. Intensities corresponding to 40% and 50% of maximal workload were calculated and used for warm-up in subsequent exercise bouts.

### Exercise bouts

All experimental exercise bouts began between 6:00 and 9:00 A.M., with starting times matched within subjects. One hour prior to arrival, participants consumed a standardized breakfast consisting of 1.5 g of carbohydrates per kilogram of body weight and were encouraged to hydrate with water. Breakfast options included a choice of a grain (oatmeal or granola bars), fruit (banana or clementines), and juice (apple or orange). The amount of carbohydrates chosen was based on Carr et al. (2011), who reported reduced GI distress with SB ingestion when preceded by a carbohydrate-rich meal and using a corn starch placebo.

Upon arrival, participants voided their bladder, and a urine sample was collected to assess hydration by measuring urine specific gravity (USG) using a refractometer (Cole-Parmer, RSA-BR90A, Vernon Hills, IL). If USG was > 1.020, the participant was considered dehydrated, and the exercise bout was rescheduled (Cheuvront et al. 2010). Following a 10-min seated rest, blood was collected from the antecubital vein (blood handling procedures are outlined below, under “blood and urine analyses”). Participants then ingested 0.2 g/kg of bodyweight of either placebo or SB in identical-looking gelatin capsules, in a double blind, randomized, counterbalanced, crossover design. Corn starch (Rumford, Terre Haute, IN, USA; Non-GMO and gluten free, containing 35 calories per 10 g) was used as a placebo, as this has been shown to be an effective placebo which does not induce GI distress Carr et al. (2011). To further reduce GI distress, ingestion was spread out into four equal doses taken every 15 min over a 45-minute period. Condition blinding was maintained by a member of the research team not involved in data collection, and blinding efficacy was assessed via an exit questionnaire at the end of the final experimental exercise bout.

After a 10-minute seated rest following ingesting the final capsule, finger prick capillary blood was collected to measure lactate using a portable lactate analyzer (Lactate Plus Analyzer, Nova Biomedical, Waltham, MA). Capillary blood was also analyzed using an i-STAT gas analyzer (CG8 + cartridge, i-STAT 1 Wireless, Abbott, Princeton, USA) for HCO_3_^−^, pH, hematocrit, and hemoglobin. Urine was again collected, and nude body weight was measured in a private room. A rectal thermistor (Level 1 esophageal/rectal temperature probe, Smiths Medical, Minneapolis, MN, USA) was self-inserted 10 cm past the anal sphincter, and a heart rate strap was strapped around the chest. The rectal thermistor was connected to a receiver (4600 precision rectal thermometer, YSI Temperature, Dayton, OH) to measure core temperature, and the heart rate strap connected to a watch (Polar H10, Kempele, Finland).

Participants entered a heat chamber set at a ~ 40 °C dry bulb temperature (placebo: 40.3 ± 1.0 °C, SB: 40.0 ± 0.9 °C) and ~ 20% relative humidity (placebo: 19% ± 9%, SB: 20% ± 9%). Baseline measurements included heart rate, core temperature, rating of perceived exertion (RPE) (Borg 1982), thermal sensation (0 – Very cold, + 8 – Very Hot), and thermal comfort (1 – Comfortable, 5 – Intolerable) (Gagge et al. 1967). GI symptoms were assessed using a 12-question visual analog questionnaire from Gaskell et al. (2019). Dry bulb temperature and relative humidity were recorded every 10 min (QUESTemp QT-44, TSI QUESTemp, Shoreview, MN).

Participants then completed a 22-min warm-up on a cycle ergometer (Lode Excalibur, Groningen, The Netherlands), consisting of 10 min of cycling at 40% and 12 min at 50% of their peak power. GI symptoms were again assessed at minute 10. At the end of minutes 20, 21, and 22, participants completed 3, 5-s self-selected standing sprints. Participants then moved to a Wingate testing cycle ergometer (Model 894E Monark, Vansbro, Sweden) and were provided with 300 mL of cool tap water (~ 12 °C). This was the only time they were allowed to drink, and the volume ingested during the first experimental exercise bouts was matched for the following exercise bout. After three minutes of seated rest, blood lactate was measured again from the finger in duplicate.

Prior to the sprint protocol, participants donned a mask connected to a metabolic cart to measure expired gases and calculate respiratory exchange ratio (RER). Metabolic gases were collected for the entirety of the sprint protocol, and RER was calculated using 15-s averages. The sprint protocol consisted of four sets of five maximal effort 6-second standing sprints. The protocol included 24 s of seated rest between each sprint, and five minutes of seated rest between sets. Resistance for the sprints was individualized to weight and adjusted to 7.5% of body mass. Sprints began when the participant reached a cadence of 110 rpm. The cycle ergometer was connected to Wingate testing software (Monark, Vansbro, Sweden), which recorded peak and average power for each sprint using a rotary encoder to measure cadence. A fan set at ~ 2.5 m/s was pointed at the torso of the participant during each set but was turned off during the 5-minute recovery period between sets. Heart rate, RPE, thermal sensation, and thermal comfort were recorded immediately after each set. Blood lactate from the finger was measured in duplicate immediately after the second and fourth sets. Symptoms of GI distress were recorded after the final set, followed by a two-minute cooldown at a self-selected intensity.

After the cooldown, participants left the heat chamber and seated rest for ten minutes before a venous blood draw. Following the blood draw, urine was collected again, and the participant measured their nude body weight. Over the next hour, participants drank a volume of water corresponding to 75% of the body weight lost during the heat exercise bout. This was followed by another blood draw and collection of urine at one-hour post-exercise. Participants returned at least seven days later to complete the alternative exercise bout.

### Blood and urine analyses

A total of ~ 42 mL of venous blood was collected per experimental exercise bout, with ~ 14 mL collected at three timepoints (pre-SB ingestion, post-exercise, and one-hour post-exercise). Venous blood from pre-ingestion and post-exercise was collected in EDTA, heparin, and serum-separating tubes, while only EDTA and serum-separating tubes were used for one-hour post-exercise. Venous blood in the heparin tubes at the pre-ingestion and post-exercise timepoints were immediately pipetted into the i-STAT gas analyzer device to measure HCO_3_^−^, pH, hematocrit, and hemoglobin. Capillary blood collected post-ingestion was analyzed in the same manner. EDTA tubes from all venous blood draws were immediately centrifuged at 1600 *g* for 10 min at room temperature to separate plasma, while the serum-separating tube sat for 30 min to allow the blood to clot before centrifugation. Plasma and serum samples were pipetted into microtubes in duplicate and stored at − 80 °C until later analyses for markers of AKI, AGI, and endotoxemia.

Time and volume of urine samples were recorded to calculate urine flow rate. USG was measured manually using a refractometer (Cole-Parmer, RSA-BR90A, Vernon Hills, IL) while urine pH was measured at all timepoints using a test strip (10-parameter urinalysis strips, Medline Industries, Northfield, IL) and urine analyzer (Model 120, Medline Industries, Northfield, IL). Urine samples were stored at − 80 °C until later analyses.

Markers of AKI were measured in urine using enzyme-linked immunosorbent assay (ELISA) kits (RayBiotech Life, Peachtree Corners, GA) for insulin-like growth factor-binding protein 7 (IGFBP7), tissue inhibitor of metalloproteinase 2 (TIMP-2), and neutrophil gelatinase-associated lipocalin (NGAL). The product of IGFBP7 and TIMP-2 (i.e. IGFBP7×TIMP-2) was calculated as this marker is the only FDA approved biomarker to predict AKI risk (Wołyniec et al. 2020). To correct for differences in urine concentration, all markers of AKI were normalized to USG (Cone et al. 2009). Plasma samples were used for other ELISA kits (Hycult Biotech, Uden, The Netherlands) to measure markers of AGI (intestinal fatty acid binding protein; I-FABP) and endotoxemia (lipopolysaccharide binding protein; LBP, and soluble cluster of differentiation 14; sCD14). All ELISA assays were run in duplicate with coefficients of variation < 10% (I-FABP, 3.2%; LBP, 9.8%; sCD14, 6.7%; IGFBP7, 6.8%; TIMP-2, 3.0%; NGAL, 9.0%). The ELISA kit manufacturers reported the following sensitivity for each respective marker: I-FABP, 47 pg/mL; LBP, 4.4 ng/mL; sCD14, 8.8 ng/mL; IGFBP7, 0.065 ng/mL; TIMP-2, 2 pg/mL; NGAL, 4 pg/mL. Plasma and urine samples were also analyzed in duplicate for osmolality (Precision Systems, Model 6002, Nattick, MA).

### Statistical analyses and calculations

Average and peak power percent decrement values for each set of five sprints (S) were calculated as follows (Girard et al. 2011; Périard et al. 2020):

1 $$\% \:\text{Decrement }\:=\:\left(1-\frac{{\sum\:}_{i=1}^{5}{S}_{i}}{{S}_{best}\times\:5}\right)\times\:100\:$$

Two-way repeated measures analysis of variance (ANOVA) tests were conducted to analyze the main effect of time, condition (placebo vs. SB), and time × condition interaction on peak and average power, peak and average power decrement, heart rate, lactate, osmolality, urine measures, RPE, thermal sensation, thermal comfort, and markers of AKI, intestinal injury, and endotoxemia. In these models, condition and time were included as within-subject factors, and participant was treated as a random effect to account for repeated measurements within individuals. Markers of AKI were log-transformed (base 10) to normalize variability, as assessed via Q-Q plots. Outliers were identified using the interquartile range (IQR) method. Specifically, values falling below the first quartile (Q1) − 1.5 × IQR or above the third quartile (Q3) + 1.5 × IQR were classified as outliers. In the instance of observed outliers, data were analyzed with and without the observed outliers. Omission of none of the observed outliers resulted in changing the significance of condition or interaction effects, so all observed outliers were retained.

Due to missing data, mixed effects models were used to compare time, condition, and interaction effects for RER, core temperature, and data from the i-STAT device (blood bicarbonate, pH, hematocrit, and hemoglobin). Linear mixed-effects models were constructed with condition (SB vs. placebo), time, and their interaction included as fixed effects, and participant included as a random intercept to account for repeated measures within individuals. For RER, two sets were missing during the second and third sets of one SB exercise bout due to the hose becoming disconnected from the mixing chamber, which was not noticed by the research team until the end of the third set. Four data points (four respective participants, all at post-SB ingestion: one SB condition, and three placebo condition) were missing from the i-STAT data due to insufficient volume of blood collected. For the core temperature data, three data points were missing from the timepoint at the end of the warm-up (two placebo, one SB) and five data points were missing from the second set of sprints timepoint (two placebo, three SB) due to the thermistor moving from the aggressive nature of 20 maximal effort standing sprints. Significant interaction effects were further explored using Bonferroni post hoc tests. Two-tailed paired t-tests were used to compare conditions for change in body weight and peak heart rate. GI symptoms were totaled for all time points and compared between conditions using a two-tailed Wilcoxon Signed-Rank test which is appropriate for non-normally distributed data. Cohen’s *d* was used to report effect size for markers of AKI, AGI, and endotoxemia. All statistical analyses and figures in the results section were created using GraphPad Prism 9.4 (GraphPad Software Inc., La Jolla, CA, USA), and a significance level of *p* ≤ 0.05 was set for all tests.

## Results

### Subject characteristics and adherence

Eleven participants were screened for this study. One participant was excluded after feelings of nausea and lightheadedness and was removed from the heat chamber following the first set of sprints during their first experimental exercise bout. Thus, statistical analyses were based on the data from 10 subjects (males = 6) (Table 1). Five of the 10 participants correctly identified the order of conditions, which is consistent with chance-level identification in a two-condition design but does not confirm effective blinding.

**Table 1:** Participant characteristics

	Males (*n* = 6)	Females (*n* = 4)	All (*n* = 10)
Age (years)	32 ± 8	35 ± 3	33 ± 7
Height (cm)	179 ± 5	170 ± 7	176 ± 7
Weight (kg)	78 ± 9	61 ± 7	71 ± 12
Body fat (%)	15.4 ± 5.2	14.7 ± 6.4	15.1 ± 5.4
V̇O_2_peak (mL/kg/min)	53.8 ± 2.7	51.1 ± 9.4	52.7 ± 6.0
Max workload (W)	374 ± 41	269 ± 18	332 ± 63

Note: V̇O_2_peak = peak oxygen uptake

### Repeated sprint ability

**Fig. 2:**
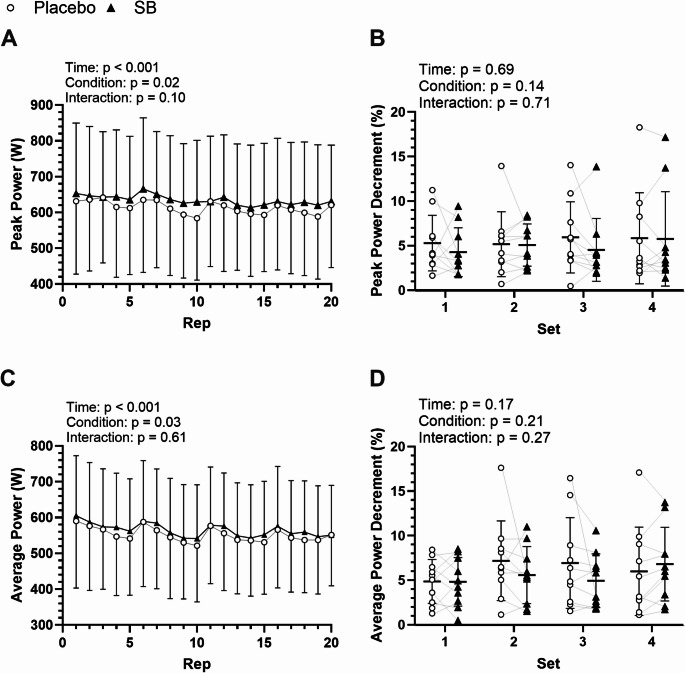
Differences between placebo (circles) and sodium bicarbonate (SB; triangles) for **A** peak power across 20 repetitions (reps), **B** percent peak power decrement for each set, **C** average power across 20 reps, and **D** percent average power decrement for each set (*n* = 10). Data are presented as mean ± standard deviation, with p-values reported for main effects of time and condition, and time x condition interaction from two-way repeated measure ANOVA analyses

There was a significant effect of condition for peak power (Fig. 2A; F_1,9_ = 8.45, *p* = 0.02), demonstrating an average of 21 W (3.4%) higher in the SB condition across the duration of the 20 sprints. There was no significant interaction effect for peak power (F_19,171_ = 1.46, *p* = 0.10), so differences between placebo and SB at specific time points (reps), were not explored. Additionally, SB had no effect on the percent peak power decrement over the four sets (Fig. 2B; effect of condition F_1,9_ = 2.60, *p* = 0.14; interaction effect F_3,27_ = 0.47, *p* = 0.71). Similarly, average power was 13 W (2.3%) higher in the SB condition (condition effect: F_1,9_ = 6.63, *p* = 0.03, Fig. 2C), reflecting higher average power with SB across the 20 sprints. There was no significant interaction effect for average power (F_19,171_ = 0.88, *p* = 0.61), so differences between placebo and SB at specific time points (reps), were not explored. There was no effect on percent average power decrement (Fig. 2D) between conditions (effect of condition F_1,9_ = 1.80, *p* = 0.21; interaction effect F_3,27_ = 1.40, *p* = 0.27).

### Physiological, thermoregulatory, and perceptual responses

**Fig. 3:**
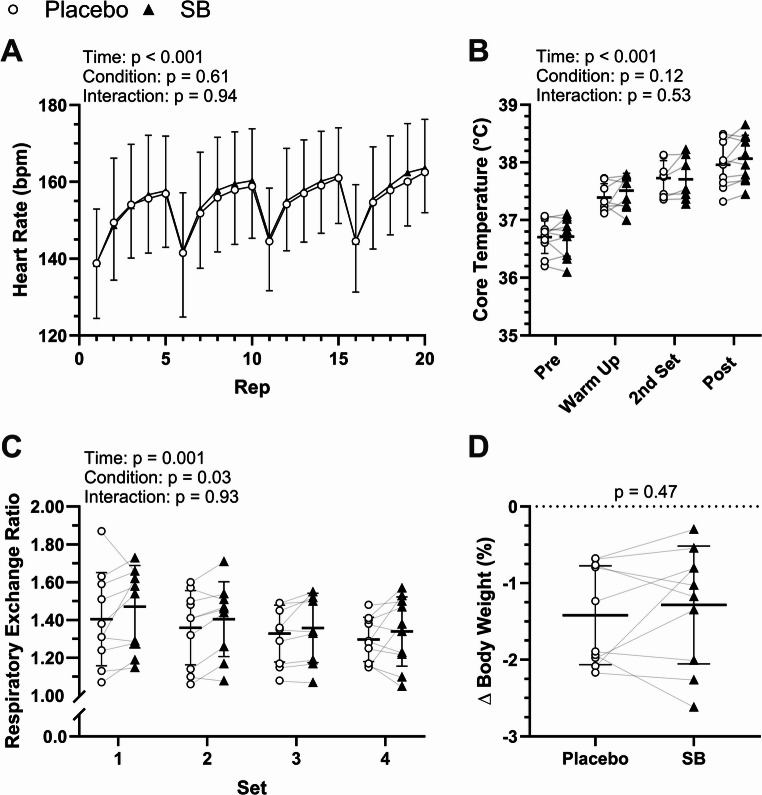
Differences between placebo (circles) and sodium bicarbonate (SB; triangles) for **A** heart rate across 20 repetitions (reps), **B** core temperature from pre-, end of warm up, the second set, and post-exercise, **C** respiratory exchange ratio for four sets, and **D** percent change in (Δ) body weight from pre- to post-exercise (*n* = 10). Data are presented as mean ± standard deviation, with p-values reported for main effects of time and condition, and time x condition interaction (**A**–**C**) or between conditions from a paired t-test (**D**). Two-way repeated measures ANOVAs were used to analyze heart rate, whereas linear mixed effects models were used due to missing data points for core temperature and respiratory exchange ratio. Differences in the change in body weight between conditions were analyzed using a paired samples t-test

There was no effect of SB on heart rate (condition effect F_1,9_ = 0.29, *p* = 0.61; interaction effect: F_19,171_ = 0.54, *p* = 0.94) or core temperature (condition effect F_1,9_ = 2.94, *p* = 0.12; interaction effect: F_3,19_ = 0.76, *p* = 0.53) (Fig. 3A and B). Peak RER averaged over the four sets was slightly higher with SB (1.39 ± 0.20) compared to placebo (1.35 ± 0.18) (condition effect: F_1,9_ = 6.58 *p* = 0.03, Fig. 3C). Change in body weight was not significantly different between placebo (-1.4% ± 0.6%) and SB (-1.3% ± 0.8%) exercise bouts (*p* = 0.47, Fig. 3D). An interaction effect (F_2,14_ =11.20, *p* = 0.001) was observed for blood HCO_3_^−^ (Table 2), however post hoc analyses revealed no significant differences at any time point (pre-ingestion: *p* = 0.47, post-ingestion: *p* = 0.06, post-exercise: *p* = 0.06). Also of note, an interaction effect (F_3,27_ = 5.15, *p* = 0.006) was observed for blood lactate with the SB condition higher after the second (*p* = 0.04) and fourth sets (*p* < 0.001) but not at baseline (*p* > 0.99) or after the warm-up (*p* > 0.99) (Table 2). There were no condition or interaction effects for blood pH, hematocrit, or hemoglobin (Table 2, *p* > 0.05).

**Table 2:** Blood markers between placebo and sodium bicarbonate (SB) conditions (*n* = 10)

Variable	Placebo	SB	F-statistic, df, and *P*-value		
			Time	Condition	Interaction
*HCO*_**3**_^**−**^, *mmol/L*			F_2,18_ =16.78	F_1,9_ =2.38	F_2,14_ =11.20
Pre-ING	24.9 ± 2.0	23.5 ± 2.6	*p* < 0.001	*p* = 0.16	*p* = 0.001
Post-ING	22.0 ± 1.6	25.8 ± 2.4			
Post-EXE	16.5 ± 3.9	18.9 ± 5.9			
*pH*			F_2,18_ =8.51	F_1,9_ =4.57	F_2,14_ =2.33
Pre-ING	7.33 ± 0.08	7.33 ± 0.14	*p* = 0.003	*p* = 0.06	*p* = 0.14
Post-ING	7.41 ± 0.03	7.46 ± 0.04			
Post-EXE	7.33 ± 0.08	7.39 ± 0.08			
*Hematocrit*,* %*			F_2,18_ =10.82	F_1,9_ =1.48	F_2,13_ =0.99
Pre-ING	44 ± 3	42 ± 5	*p* < 0.001	*p* = 0.26	*p* = 0.40
Post-ING	47 ± 4	48 ± 6			
Post-EXE	45 ± 3	44 ± 4			
*Hemoglobin*,* g/dL*			F_2,18_ =11.16	F_1,9_ =1.64	F_2,13_ =1.05
Pre-ING	14.9 ± 0.9	14.4 ± 1.5	*p* < 0.001	*p* = 0.23	*p* = 0.38
Post-ING	16.1 ± 1.3	16.4 ± 1.9			
Post-EXE	15.3 ± 1.2	14.9 ± 1.2			
*Lactate*,* mmol/L*			F_3,27_ =63.38	F_1,9_ =9.63	F_3,27_ =5.15
Pre-EXE	1.9 ± 1.0	1.8 ± 0.8	*p* < 0.001	*p* = 0.01	*p* = 0.006
Post-warm up	4.2 ± 1.8	4.1 ± 1.5			
Post-2nd Set	10.9 ± 4.2	12.8 ± 4.0*			
Post 4th Set	11.1 ± 5.0	14.1 ± 3.9*			
*Plasma Osmolality*,* mOsm*			F_2,18_ =1.07	F_1,9_ =2.19	F_2,18_ =1.17
Pre-ING	300 ± 12	300 ± 10	*p* = 0.36	*p* = 0.17	*p* = 0.33
Post-EXE	302 ± 9	308 ± 17			
1 H Post-EXE	300 ± 10	304 ± 4			

Data are presented as mean ± standard deviation, with p-values reported for main effects of time and condition, and time x condition interaction. Two-way repeated ANOVAs were conducted for lactate and plasma osmolality, whereas mixed effects models were used for the other variables due to missing data points. Capillary blood was used for blood lactate measurements, and venous blood was used to measure plasma osmolality. For the other variables, venous blood was used pre-ingestion and post-exercise, whereases capillary blood was used post-ingestion

Note: ING – ingestion, EXE – exercise, HCO_3_^−^ – bicarbonate, * - indicates significant difference at same time point from placebo condition (*p* ≤ 0.05)

Perceptual responses (RPE, thermal sensation, thermal comfort) are shown in Table 3. RPE increased (time effect: F_4,36_ =407.9, *p* < 0.001) throughout the sprint protocol, but there were no condition (F_1,9_ =1.20, *p* = 0.30) or interaction (F_4,36_ =0.57, *p* = 0.69) effects. Similar results were found for thermal sensation and thermal comfort time effects (thermal sensation: F_4,36_ =17.40, *p* < 0.001, thermal comfort: F_4,36_ =47.68, *p* < 0.001), but no effects of condition (thermal sensation: F_1,9_ =1.03, *p* = 0.34, thermal comfort: F_1,9_ =0.25, *p* = 0.63) or interaction (thermal sensation: F_4,36_ =2.13, *p* = 0.10, thermal comfort: F_3,25_ =0.20, *p* = 0.93).

**Table 3:** Perceptions of exertion and heat stress between placebo and sodium bicarbonate (SB) conditions (*n* = 10)

Variable	Placebo	SB	F-statistic, df, and *P*-value		
			Time	Condition	Interaction
*RPE*			F_4,36_ =407.9	F_1,9_ =1.20	F_4,36_ =0.57
Baseline	6 ± 0	6 ± 0	*p* < 0.001	*p* = 0.30	*p* = 0.69
Set 1	16 ± 1	15 ± 2			
Set 2	17 ± 1	16 ± 1			
Set 3	18 ± 1	18 ± 1			
Set 4	18 ± 1	18 ± 2			
*Thermal sensation*			F_4,36_ =17.40	F_1,9_ =1.03	F_4,36_ =2.13
Baseline	5 ± 1	5 ± 1	*p* < 0.001	*p* = 0.34	*p* = 0.10
Set 1	5 ± 1	5 ± 1			
Set 2	7 ± 1	6 ± 1			
Set 3	6 ± 1	6 ± 1			
Set 4	7 ± 1	7 ± 1			
*Thermal comfort*			F_4,36_ =47.68	F_1,9_ =0.25	F_3,25_ =0.20
Baseline	1 ± 0	1 ± 0	*p* < 0.001	*p* = 0.63	*p* = 0.93
Set 1	2 ± 1	2 ± 1			
Set 2	3 ± 1	3 ± 1			
Set 3	3 ± 1	3 ± 1			
Set 4	3 ± 1	3 ± 1			

Data are presented as mean ± standard deviation, with p-values from two-way repeated measures ANOVAs reported for main effects of time and condition, and time x condition interaction

Note: RPE – Rating of perceived exertion

### Markers of acute kidney injury

**Fig. 4:**
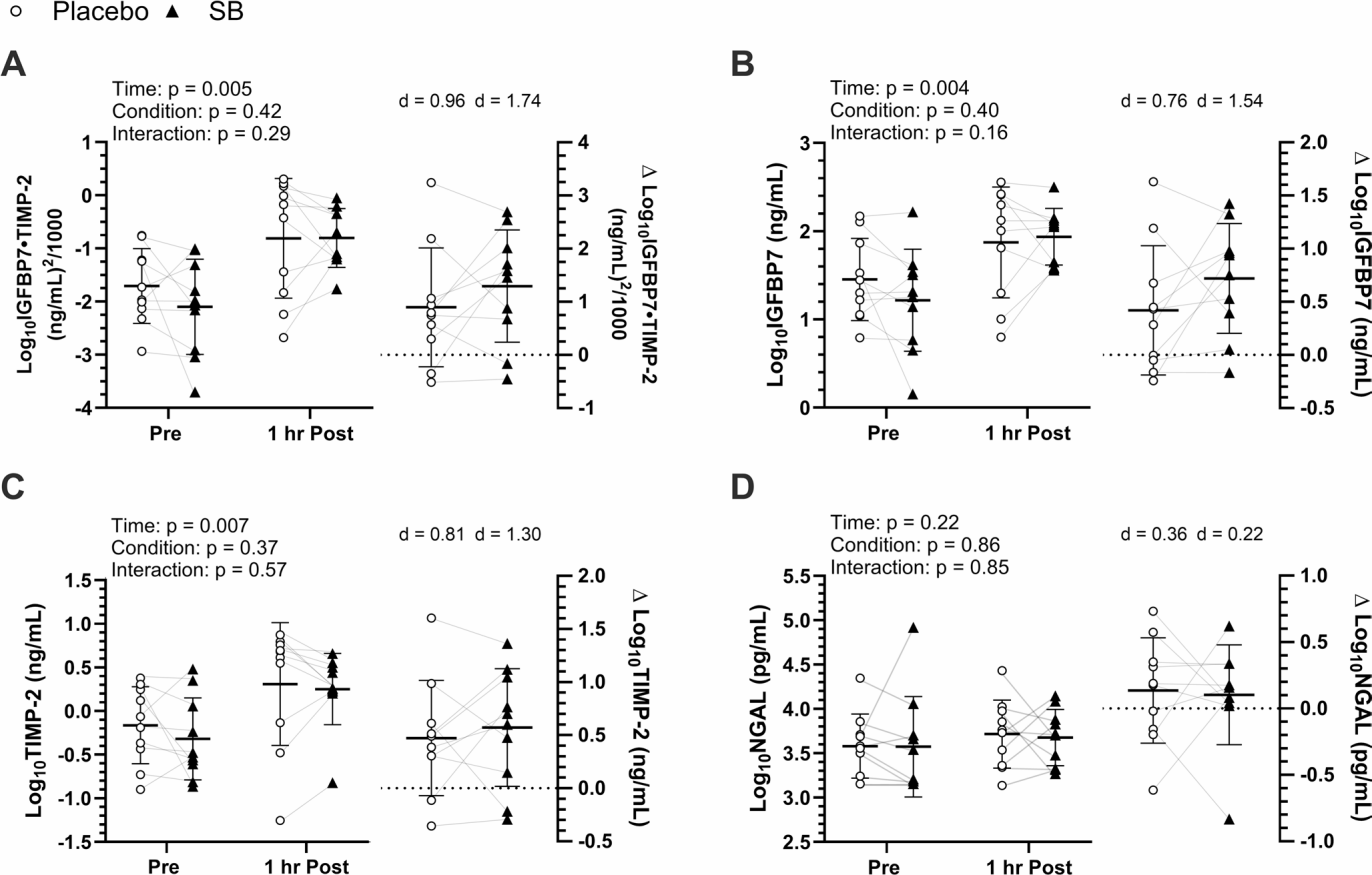
Differences between placebo (circles) and sodium bicarbonate (SB; triangles) for urinary markers of acute kidney injury (*n* = 10). Markers reported include (**A**) the mathematical product of urinary insulin-like growth factor-binding protein 7; IGFBP7 and urinary tissue inhibitor of metalloproteinase-2; TIMP-2 (IGFBP7×TIMP-2), (**B**) IGFBP7, (**C**) TIMP-2, and (**D**) neutrophil gelatinase-associated lipocalin (NGAL). The left panel shows values at pre- to 1-hour post-exercise with p-values from two-way repeated measures ANOVAs reported for main effects of time and condition, and time x condition interaction. The right panel shows the change in (Δ) these values from pre- to 1-hour post-exercise and displays effect size as Cohen’s *d*. Data are presented as mean ± standard deviation. Markers were normalized to urine specific gravity and log (base ten) transformed due to non-normal distributions

Urine pH, USG, and urine flow rate are presented in Table 4. Briefly, urine pH showed an interaction effect (F_3,27_ =19.7, *p* < 0.001), which was higher in the SB condition post-ingestion (*p* = 0.001), post-exercise (*p* < 0.001), and one-hour post-exercise (*p* < 0.001), but not different at baseline (*p* > 0.99). USG demonstrated a main effect for time (F_3,27_ =9.37, *p* < 0.001), with no condition (F_1,9_ =2.19, *p* = 0.17) or interaction (F_3,27_ =0.21, *p* = 0.89) effects. Similarly, urine flow rate showed a time effect (F_3,27_ =6.98, *p* < 0.001), but no condition (F_1,9_ =0.07, *p* = 0.80) or interaction (F_3,27_ =0.20, *p* = 0.89) effects. Our primary marker of AKI, urinary IGFBP7×TIMP-2 (Fig. 4A), increased from pre- to one-hour post-exercise (time effect: F_1,9_ = 13.72, *p* = 0.005, placebo *d* = 0.96, SB *d* = 1.74). However, there was no condition (F_1,9_ = 0.71, *p* = 0.42) or interaction (F_1,9_ = 1.29, *p* = 0.29) effect. When exploring IGFBP7 (Fig. 4B) and TIMP-2 (Fig. 4C) independently, we found a similar time effect (IGFBP7: F_1,9_ = 14.64, *p* = 0.004, placebo *d* = 0.76, SB *d* = 1.54; TIMP-2: F_1,9_ = 11.89, *p* = 0.007, placebo *d* = 0.81, SB *d* = 1.30) where the markers increased from pre- to one-hour post-exercise. There were no condition (IGFBP7: F_1,9_ = 0.49, *p* = 0.40; TIMP-2: F_1,9_ = 0.89, *p* = 0.37) or interaction (IGFBP7: F_1,9_ = 2.31, *p* = 0.16; TIMP-2: F_1,9_ = 0.34, *p* = 0.57) effects. When exploring the non-log transformed data at one-hour post-exercise, six participants (60%) in the placebo condition and four (40%) in the SB condition had an IGFBP7×TIMP-2 concentration of greater than 0.3 (ng/mL)^2^/1000, which is a clinical threshold indicative of elevated risk of developing AKI in the following 12 h (Hoste et al. 2014). Lastly, we did not find any time (F_1,9_ = 1.72, *p* = 0.22, placebo *d* = 0.36, SB *d* = 0.22), condition (F_1,9_ = 0.03, *p* = 0.86), or interaction (F_1,9_ = 0.04, *p* = 0.85) effects for urinary NGAL (Fig. 4D).

**Table 4:** Urine pH, specific gravity (USG), and flow rate between placebo and sodium bicarbonate (SB) conditions (*n* = 10)

Variable	Placebo	SB	F-statistic, df, and *P*-value		
			Time	Condition	Interaction
*Urine pH*			F_3,27_ =10.75	F_1,9_ =34.38	F_3,27_ =19.77
Pre-ING	6.1 ± 0.2	6.2 ± 0.5	*p* < 0.001	*p* < 0.001	*p* < 0.001
Post-ING	6.1 ± 0.2	6.9 ± 0.9*			
Post-EXE	6.0 ± 0.2	7.6 ± 0.8*			
1 H Post-EXE	6.0 ± 0.2	8.1 ± 1.1*			
*USG*			F_3,27_ =9.37	F_1,9_ =2.19	F_3,27_ =0.21
Pre-ING	1.012 ± 0.006	1.012 ± 0.009	*p* < 0.001	*p* = 0.17	*p* = 0.89
Post-ING	1.008 ± 0.008	1.010 ± 0.007			
Post-EXE	1.012 ± 0.006	1.014 ± 0.006			
1 H Post-EXE	1.018 ± 0.007	1.020 ± 0.004			
*Urine flow Rate*,* mL/h*			F_3,27_ =6.98	F_1,9_ =0.07	F_3,27_ =0.20
Pre-ING	156 ± 60	181 ± 105	*p* < 0.001	*p* = 0.80	*p* = 0.89
Post-ING	360 ± 314	351 ± 278			
Post-EXE	117 ± 64	122 ± 75			
1 h Post-EXE	71 ± 53	77 ± 26			
*Urine Osmolality*,* mOsm*			F_2,18_ =12.10	F_1,9_ =1.19	F_2,18_ =2.30
Pre-ING	405 ± 209	370 ± 245	*p* < 0.001	*p* = 0.30	*p* = 0.13
Post-EXE	330 ± 189	478 ± 196			
1 H Post-EXE	607 ± 220	662 ± 107			

Data are presented as mean ± standard deviation, with p-values from two-way repeated measures ANOVAs reported for main effects of time and condition, and time x condition interaction

SB – Sodium bicarbonate, USG – Urine specific gravity

### Gastrointestinal distress, acute gastrointestinal injury, and endotoxemia

**Fig. 5:**
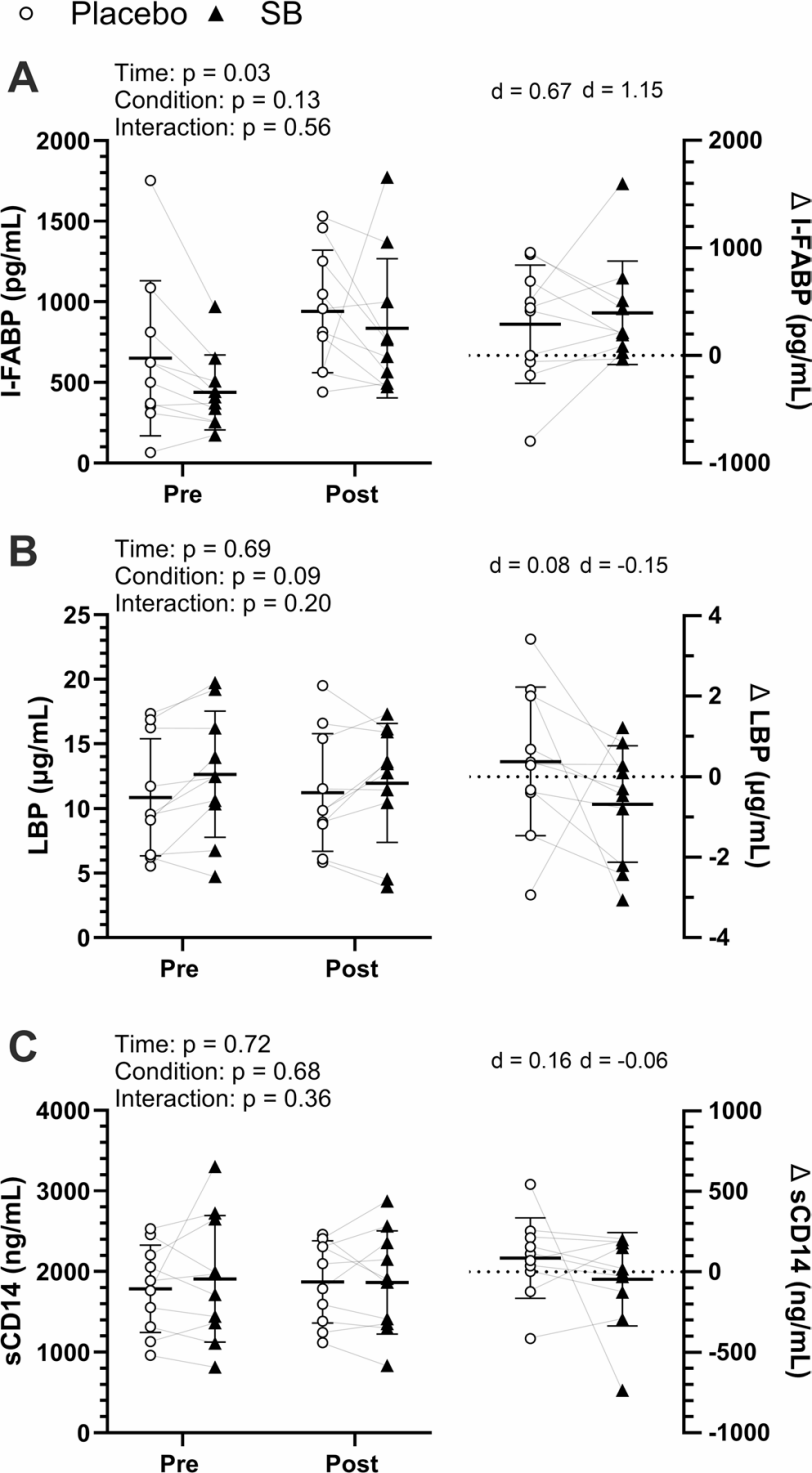
Differences between placebo (circles) and sodium bicarbonate (SB; triangles) for plasma markers of acute gastrointestinal injury (**A** intestinal fatty acid binding protein; I-FABP) and endotoxemia (**B** lipopolysaccharide binding protein; LBP, and** C** soluble cluster of differentiation 14; sCD14) (*n* = 10). The left panel shows values at pre- to immediate post-exercise with p-values from two-way repeated measures ANOVAs reported for main effects of time and condition, and time x condition interaction. The right panel shows the change in (Δ) these values from pre- to post-exercise and displays effect size as Cohen’s *d*. Data are presented as mean ± standard deviation

Despite GI distress being one of the most reported side effects of SB supplementation, no differences were observed between conditions for upper (*p* = 0.58), lower (*p* = 0.25), other (*p* = 0.75), or overall (*p* = 0.23) GI symptoms (Table 5). A time effect was observed (F_1,9_ = 6.17, *p* = 0.03, placebo *d* = 0.67, SB *d* = 1.15) for I-FABP (Fig. 5A), a marker of AGI, which increased from pre- to post-exercise, with no condition (F_1,9_ = 2.70, *p* = 0.13) or interaction (F_1,9_ = 0.37, *p* = 0.56) effect. For markers of endotoxemia, neither LBP nor sCD14 demonstrated significant effects of time (LBP: F_1,9_ = 0.17, *p* = 0.69, placebo *d* = 0.08, SB *d* = -0.15; sCD14: F_1,9_ = 0.14, *p* = 0.72, placebo *d* = 0.16, SB *d* = -0.06), condition (LBP: F_1,9_ = 3.73, *p* = 0.09; sCD14: F_1,9_ = 0.18, *p* = 0.68), or interaction (LBP: F_1,9_ = 1.95, *p* = 0.20; sCD14: F_1,9_ = 0.95, *p* = 0.36).

**Table 5:** Gastrointestinal symptom scores for placebo and sodium bicarbonate (SB) conditions (*n* = 10)

Symptoms	*Placebo*		*SB*		*P*-Value
	Incidence	Median (min, max)	Incidence	Median (min, max)	
Upper	50%	2 (0, 6)	60%	1 (0, 9)	0.58
Lower	10%	0 (0, 3)	30%	0 (0, 6)	0.25
Other	20%	0 (0, 2)	10%	0 (0, 1)	0.75
Overall	50%	2 (0, 6)	60%	3 (0, 12)	0.23

Data are presented as percent incidence of gastrointestinal symptoms (score of ≥ 1), with the median and range (min, max) of scores totaled over three time points. P-values from Wilcoxon signed-rank tests comparing experimental conditions are also reported

## Discussion

The aims of this study were to test the hypotheses that SB supplementation would: (1) improve performance, (2) attenuate increases in markers of AKI, and (3) exacerbate AGI and endotoxemia during repeated sprints in a hot environment. Consistent with our hypotheses, SB improved RSA, indicated by higher peak and average power. However, SB did not affect GI symptoms or markers of AKI, AGI, or endotoxemia. Taken together, these findings provide evidence that SB, when taken after a high-carbohydrate meal, is an effective ergogenic aid for repeated high-intensity performance in the heat without exacerbating heat strain, AKI, or AGI.

We observed a significant increase in peak power and average power over 20 sprints in the heat after SB ingestion, reflecting greater total mechanical output. Surprisingly, we did not observe differences between conditions for the percent of decrement, indicating that there was not a difference in the relative decline in performance across repeated efforts within each set. These findings demonstrate that the ergogenic effects in the present study may have been related to enhanced excitability of muscle firing and increased rate of force development (Siegler et al. 2013) rather than solely attenuating fatigue from metabolic acidosis. We also observed higher lactate concentrations, RER, and urinary pH in the SB condition, indicating that SB likely improved performance through greater extracellular buffering. In the blood, HCO_3_^−^ buffers excess hydrogen ions, which may or may not alter pH depending on the influence of numerous other determinants of cellular and blood acid-base-balance (Péronnet et al. 2007). We speculate that elevated efflux of hydrogen ions (via co-transportation with lactate via monocarboxylate transporters) attenuated intramuscular metabolic acidosis, which occurs during high-intensity exercise in the heat (Febbraio 2001; Mitchell et al. 2014) and reduces performance by interfering with enzyme activity, cross-bridge cycling, and intramuscular calcium release (Cairns and Lindinger 2025). To our knowledge, the only previous study to investigate the effects of SB on sprint performance in the heat was by Mündel (2018). While they did show that SB improved the second of two Wingate tests, the study was limited in that they only completed two maximal effort sprints (Mündel 2018). Altogether, we provide evidence that SB is an effective aid to increase RSA in the heat for recreationally endurance-trained individuals, likely by attenuating intramuscular metabolic acidosis through greater extracellular buffering (higher efflux of lactate and hydrogen ions).

A concern of SB supplementation use in the heat is the potential increase in physiological and/or thermoregulatory strain, which could impair performance and increase the risk of heat-related illness. Indeed, our research group recently demonstrated that SB ingestion prior to a two hour outdoor simulated work session in the heat (dry bulb: ~35 °C, relative humidity: ~20%) increased both heart rate (mean increase of 9 ± 3 bpm) and core temperature (mean increase of 0.2 ± 0.1 °C) compared to placebo (Siegler et al. 2025), although lower RPE was observed (mean difference of 0.3 ± 0.1). Conversely, the present study observed no effects of SB on heart rate, core temperature, RPE, thermal sensation, or thermal comfort between conditions. These discrepancies are likely due to methodological differences in exercise intensity, duration, and type. Importantly, our findings support that SB improves RSA performance without worsening heat strain or altering perceptual responses.

Exertional heat stress also places considerable strain on the kidneys and can lead to AKI in extreme cases (Schlader et al. 2019). AKI associated with exertional heat stress is often reversible and is characterized by a quick decline in kidney function, largely observed by reductions in glomerular filtration rate (Basile et al. 2012). Proposed mechanisms contributing to AKI include dehydration, hyperthermia, sympathetic nerve activity, and/or muscle damage, which contribute to the depletion of ATP via reduced production and/or increased demand (Schlader et al. 2019; Masoud et al. 2024). In response to exertional heat stress, it has also been proposed that these mechanisms may shift to more glycolytic pathways due to limited oxygen supply (Masoud et al. 2024). If so, SB could be a beneficial nutritional aid in reducing the risk of AKI by extracellular buffering and mitigating metabolic acidosis at the site of the kidney. Indeed, Sánchez-Lozada et al. (2018) demonstrated that ingesting SB after prolonged heat exposure and repeated bouts of muscle damage pointedly reduced markers of AKI in rats. In humans, we recently reported that SB attenuated the increase in IGFBP7×TIMP-2 in response to two hours of low-intensity (65% of maximal heart rate) walking in the heat (dry bulb: 40 °C, relative humidity: ~20%), with a more pronounced effect on TIMP-2 than on IGFBP7 (Masoud et al. 2025). In the present study, while SB did not attenuate concentrations of markers of AKI, fewer participants in the SB group surpassed the clinical IGFBP×TIMP-2 threshold for elevated AKI risk compared to placebo. These findings suggest a potentially meaningful difference in AKI risk, which warrants further studies with larger sample sizes to investigate the potential protective effect of SB on heat-related AKI.

Although the focus regarding SB and sport performance is centered around HCO_3_^−^, the increased sodium ingested with SB likely impacts hydration status, particularly during exercise in hot conditions. Indeed, sodium ingestion prior to exercise is known to increase plasma osmolality, reduce urine output, and improve retention of fluid (Stachenfeld 2008; Savoie et al. 2015). Surprisingly, we did not observe differences in any of these variables, nor did we see differences in hematocrit, hemoglobin, or urine osmolality. The results may be due to competing renal responses to sodium and HCO_3_^−^. While high sodium in the blood promotes fluid retention by upregulating the renin-angiotensin-aldosterone system, high blood pH contributes to renal compensation, where HCO_3_^−^ is quickly excreted through urine (Lindinger et al. 2000). Notably, we did observe a quick increase in urine pH by the post-ingestion/pre-exercise time point, demonstrating that some of the HCO_3_^−^ ingested was already being excreted by the kidneys. Likely, this renal compensation counteracted the fluid retention effects of sodium ingestion.

It is well documented that SB supplementation increases GI distress, typically peaking ~ 90 min after ingestion (Carr et al. 2011). In the present study, we demonstrate that 0.2 g/kg of SB after a high-carbohydrate meal did not worsen symptoms of GI distress and, for the first time, show that SB did not exacerbate exercise heat stress-induced markers of AGI (I-FABP) or endotoxemia (LBP, sCD14). Endotoxemia is of particular concern during exertional heat stress, as this is one of the major drivers contributing to the often-fatal outcome of exertional heat stroke (Armstrong et al. 2018). Our findings suggest that, under these conditions (moderate SB dose, co-ingestion with carbohydrate, and limited dehydration), SB does not impose an additional burden on the GI tract during high-intensity exercise in the heat. Further research should explore whether endotoxemia occurs concomitantly when GI distress is induced by SB ingestion under different dosing strategies or environmental and exercise stressors.

While our findings are novel, this study has several limitations. First, the sample size (*n* = 10) may not have been large enough to detect small to moderate effects, particularly for AKI and GI biomarkers. Second, participants were young, recreationally endurance-trained adults, so our results may not be applicable to other populations, which restricts generalizability to other populations (e.g., workers, older adults). Also, the magnitude of adaptations related to acid-base regulation and lactate transport may differ from those observed in elite athletes which may influence the generalizability of these findings. Third, participants did not experience significant dehydration, which may have limited stress to the kidneys and guts. Additionally, we had missing data for some variables (core temperature, RER, i-STAT variables) which resulted in using mixed effects models rather than two-way repeated measures ANOVAs, which may have increased the risk for a type I error. Finally, we did not control for menstrual cycle, which could alter thermoregulatory outcomes. However, Christison et al. (2025) found that while mid-luteal phase increased baseline core temperature, menstrual status did not impact the rate of change in core temperature during heat stress. Since we did not observe any difference in baseline core temperature between groups, the effect of menstrual status was likely minimal in this study.

## Conclusions

This was the first study to investigate the impact of SB on performance, and markers of AKI, AGI, and endotoxemia during repeated sprints in the heat. SB supplementation, taken after a high-carbohydrate meal, is an effective strategy to improve high-intensity exercise performance in the heat without increasing dehydration, hyperthermia, AKI risk, and GI distress or injury. Future research should explore whether these findings remain similar during periods of greater dehydration, longer exercise durations, and/or more extreme heat stress.

## Data Availability

The data that support the findings of this study are available from the corresponding author, JWS, upon reasonable request.

## Abbreviations

AGI: Acute gastrointestinal injury
AKI: Acute kidney injury
ANOVA: Analysis of variance
ELISA: Enzyme–linked immunosorbent assay
FDA: Food and drug administration
GI: Gastrointestinal
HCO_3_^−^: Bicarbonate
I-FABP: Intestinal fatty acid binding protein
IGFBP7: Insulin–like Growth Factor–binding Protein 7
LBP: Lipopolysaccharide Binding Protein
NGAL: Neutrophil Gelatinase–associated Lipocalin
PAR-Q: Physical activity readiness questionnaire
RER: Respiratory exchange ratio
RPE: Rating of perceived exertion
RSA: Repeat sprint ability
SB: Sodium bicarbonate
sCD14: Soluble cluster of differentiation 14
Tc: Core temperature
TIMP-2: Tissue Inhibitor of Metalloproteinase 2
USG: Urine specific gravity
V̇O_2_peak: Peak oxygen consumption
